# Supplementary material for: Machine Learning-Based Prediction of Glioma IDH Gene Mutation Status Using Physio-Metabolic MRI of Oxygen Metabolism and Neovascularization (A Bicenter Study)
Source: Cancers (Basel). 2024 Mar 8;16(6):1102. doi: 10.3390/cancers16061102 (PMC10969299; doi:10.3390/cancers16061102)
Supplement: Supplementary file 1 [file cancers-16-01102-s001.zip › cancers-2872101-supplementary.pdf]

Supplementary Materials

1. Patients

Supplementary Table S1. Patient Characteristics of the Cohorts.

Site	Cohort	Investigation period	Number of patients	Sex [f / m]	Age [years]	IDHwt gliomas	IDHmut gliomas
St. Pölten	Training	January 2016 to January 2023	166	81 / 85	58.1 ± 15.2 (19 – 89)	123	43
St. Pölten	Independent Internal Testing	February 2023 to May 2023	16	8 / 8	51.9 ± 18.6 (19 –76)	12	4
Erlangen	Independent External Testing	June 2016 to October 2018	33	7 / 26	54.7 ± 15.3 (27 –78)	21	12

Note. – The age of the patients is the mean value ± standard deviation, the age range is in parentheses.

2. MRI data Acquisition

Supplementary Table S2. Sequence parameters of the MRI study protocols at the University Clinic St. Pölten and the Friedrich-Alexander University Erlangen-Nürnberg.

Sequence	Site	In-plane resolution	Slice thickness [mm]	Number of slices	TR [ms]	TE [ms]	Flip angle* [°]	other
FLAIR	St. Pölten	0.45 × 0.45	3	48	5000	460	120	TI = 1800 ms
	Erlangen	0.45 × 0.45	4.5	24	10000	83	150	TI = 1600 ms
CE T1w	St. Pölten	1.0 × 1.0	1	50	2100	2.3	12	
	Erlangen	0.9 × 0.9	4.8	12	300	2.4	70	
DWI	St. Pölten	1.2 × 1.2	4	29	5300	98	90	b = 0 and 1000 s/mm2
	Erlangen	1.8 × 1.8	4	8	1500	81	90	b = 0 and 1000 s/mm2
GE-DSC	St. Pölten	1.8 × 1.8	4	29	1740	22	90	60 dynamic volumes
SE-DSC	St. Pölten	1.8 × 1.8	4	29	1740	33	90	60 dynamic volumes
GESE-DSC	Erlangen	1.8 × 1.8	4	8	1380	GE: 16; SE: 89	90	80 dynamic volumes
T2 mapping	St. Pölten	1.8 × 1.8	4	29	3260	13 – 104	90	8 echoes
	Erlangen	1.8 × 1.8	4	8	1610	12 – 96	90	8 echoes
T2* mapping	St. Pölten	1.8 × 1.8	4	29	1210	5 – 40	90	8 echoes
	Erlangen	1.8 × 1.8	4	8	658	5 – 40	90	8 echoes

Note. FLAIR, fluid-attenuated inversion-recovery; CE T1w, contrast-enhanced T1-weoghted MRI; DWI, diffusion-weighted imaging; GE-DSC, gradient echo dynamic susceptibility contrast perfusion MRI; SE-DSC, spin echo dynamic susceptibility contrast perfusion MRI; GESE-DSC, hybrid single-shot gradient-echo spin-echo dynamic susceptibility contrast perfusion MRI. *Flip angle means the angle of excitation. Refocusing angles were 180° for all sequences with a SE scheme, i.e. FLAIR, DWI, SE-DSC, and T2 mapping.

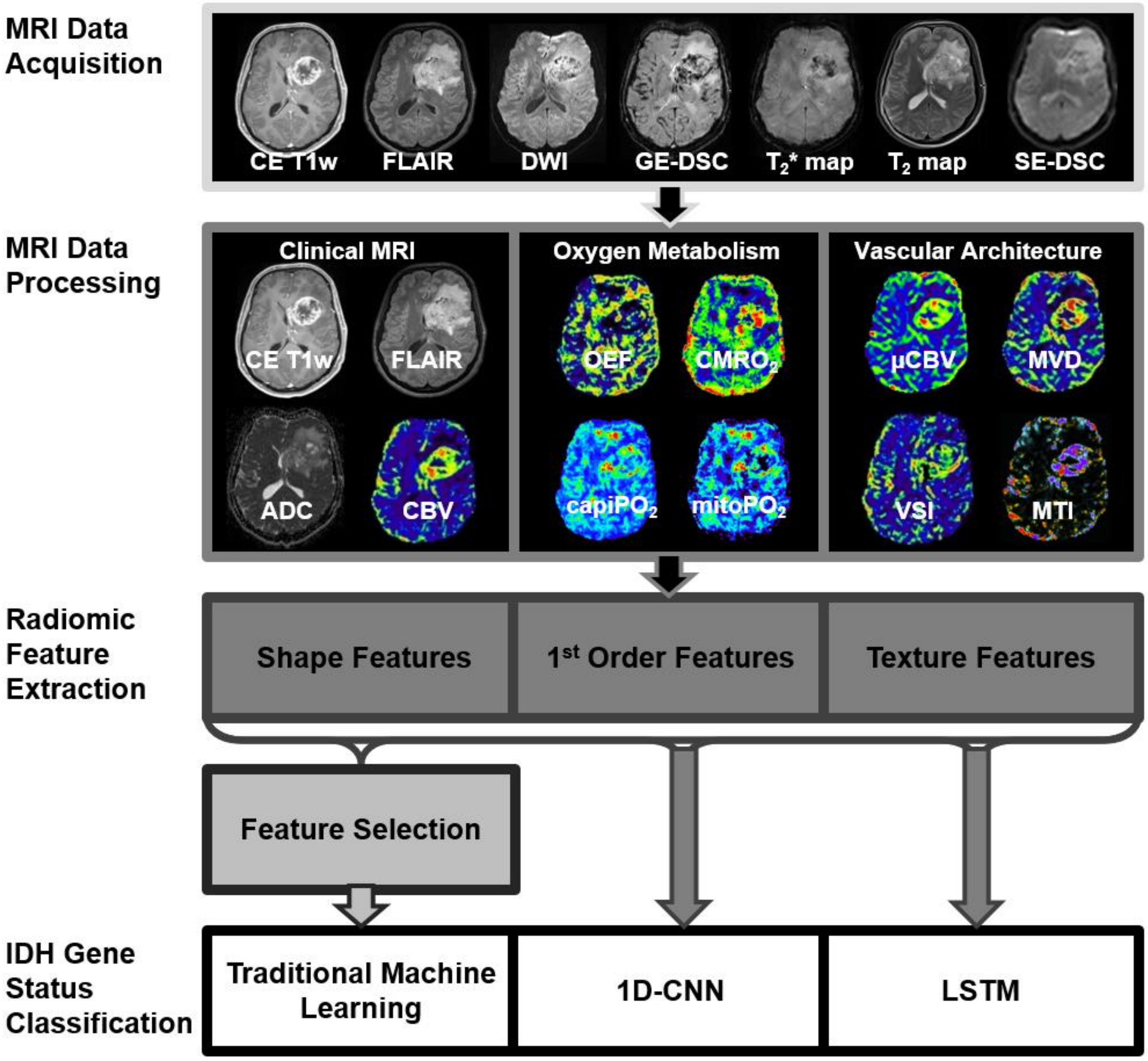

Supplementary Figure S1: The overall study pipeline.

CE T1w = contrast enhanced T1-weighted; FLAIR = fluid-attenuated inversion-recovery; DWI = diffusion-weighted imaging; GE-DSC = gradient echo dynamic susceptibility contrast perfusion MRI; SE-DSC = spin echo dynamic susceptibility contrast perfusion MRI; ADC = apparent diffusion coefficient; CBV = cerebral blood volume; OEF = oxygen extraction fraction; $CMRO_2$ = cerebral metabolic rate of oxygen; $capiPO_2$ = capillary oxygen tension; $mitoPO_2$ = mitochondrial oxygen tension; µCBV = microvascular cerebral blood volume; MVD = microvessel density; VSI = vessel size index; MTI = microvessel type indicator; CNN = Convolutional Neural Networks; LSTM = Long short-term memory.

3. Radiomic Feature Extraction

The data for clinical MRI (CE T1w FLAIR, ADC and CBV), MRI-based oxygen metabolism (OEF, $CMRO_2$, capiPO_2, and mitoPO_2), MRI-based vascular architecture (µCBV, MVD, VSI), and MRI-based neovascularization activity (MTI) of a patient were loaded into the open-source software platform 3D Slicer (v. 4.11) and aligned geometrically. Segmentation of the tumor volume was performed on CE T1w MRI data defined as the contrast-enhancing areas. Segmentation of the peritumoral edema was performed on FLAIR data defined as hyperintense areas excluding the contrast-enhancing or necrotic portions. The regions of interest (ROIs) were manually drawn on all axial slices showing the features for 3D segmentation by a MR physicist (A.S., with 22 years of experience in neuro-oncological imaging) and confirmed by another neurosurgeon (F.M., with 16 years of experience). Disagreements were resolved by discussion to consensus. Both readers were blinded to the histopathological diagnosis of the tumor.

Grey-level intensity values of the cMRI were normalized by subtracting the mean intensity and dividing by the standard deviation with an expected resulting range [-3, 3], a mean of 0 and standard deviation of 1 in the normalized image. This procedure is also known as z-score normalization [1,2]. The grey-level discretization was done [3] with a bin width value of 0.1 resulting in histograms with approximately 60 bins. Biomarker maps for both clinical MRI and physio-metabolic MRI represented quantitative imaging data with a range of physiological reasonable values. Individually adapted thresholds were applied to the biomarker maps in order to remove non-physiological values due to imaging artefacts (e.g. motion or susceptibility artefacts). Biomarker value discretization was performed with adapted bin width values in order to obtain histograms with 60 – 67 bins. **Supplementary Table 3** summarizes the value ranges and bin sizes used for the biomarker maps. Next, MRI data were resampled into a uniform voxel size of 1 × 1 × 1 mm^3 across all patients [3].

Supplementary Table S3. Value ranges, bin sizes, and bin numbers for discretization of biomarker maps.

	**ADC**	**CBV**	**OEF**	**$CMRO_2$**	**xPO_2**	**µCBV**	**MVD**	**VSI**	**MTI**
**range**	[0,3]	[0,100]	[0,100]	[0,1000]	[0,200]	[0.30]	[0,2000]	[0,500]	[-1000, 1000]
**unit**	mm^2/s	%	%	µmol/ 100g×min	mmHg	%	mm^{-2}	µm	$s^{-2/5}$
**bin size**	0.05	1.5	1.5	15	3	0.5	30	8	30
**bins**	60	67	67	67	67	60	67	63	67

Abbreviations. ADC = apparent diffusion coefficient; CBV = cerebral blood volume; µCBV = microvascular cerebral blood volume; MVD = microvessel density; VSI = vessel size index; MTI = microvessel type indicator; OEF = oxygen extraction fraction; $CMRO_2$ = cerebral metabolic rate of oxygen; and xPO_2 = cpaiPO_2 and mitoPO_2 = capillary and mitochondrial oxygen tension, respectively.

Radiomic features were extracted with the built-in package SlicerRadiomics implemented in the 3D Slicer platform based on the Python package PyRadiomics [4]. Procedures and features were in accordance with the Imaging Biomarker Standardization Initiative (IBSI) [5]. The following features were calculated:

- 14 shape features, which represent the three-dimensional size and shape of the segmented volume of interest (VOI, i.e. contrast-enhancing tumor and peritumoral edema). These features included elongation, flatness, least and major axis length, maximum 2D diameter column, maximum 2D diameter row, maximum 2D diameter slice, maximum 3D diameter, mesh volume, minor axis length, sphericity, surface area, surface volume ratio, and voxel volume.

- 18 first-order features, which represent the distribution of gray values within an image, were calculated from the histogram of voxel intensities. These features included 10th and 90th percentile, energy, entropy, interquartile range, kurtosis, maximum, mean absolute deviation, mean, median, minimum, range, robust mean absolute deviation, root mean squared, skewness, total energy, uniformity, and variance.

- 75 texture features, which describe relationships between neighboring voxels with similar or dissimilar values. These features included the following 6 subcategories: (**i**) 24 gray-level co-occurrence matrix (GLCM) features characterizing how often pairs of voxels with specific intensity levels and spatial relationship occur in an image [6]; (**ii**) 14 gray-level dependence matrix (GLDM) features representing the dependency of connected voxels to a center voxel [7]; (**iii**) 16 gray-level run-length matrix (GLRLM) features evaluating the length of consecutive pixels with the same gray level [8]; (**iv**) 16 gray-level size zone matrix (GLSZM) features quantifying the number of connected voxels that share the same intensity value [9]; and (**v**) five neighboring gray-tone difference matrix (NGTDM) features assessing differences between pixel values and neighbor average gray value [10].

Supplementary Table S4. List of radiomic features.

**Shape Features**	Elongation, Flatness, Least Axis Length, Major Axis Length, Maximum 2D Diameter Column, Maximum 2D Diameter Row, Maximum 2D Diameter Slice, Maximum 3D Diameter, Mesh Volume, Minor Axis Length, Sphericity, Surface Area, Surface Volume Ratio, Voxel Volume
**First-Order Features**	10th Percentile, 90th Percentile, Energy, Entropy, Interquartile Range, Kurtosis, Maximum, Mean Absolute Deviation, Mean, Median, Minimum, Range, Robust Mean Absolute Deviation, Root Mean Squared, Skewness, Total Energy, Uniformity, Variance
**Texture Features**	**GLCM:** Autocorrelation, Cluster Prominence, Cluster Shade, Cluster Tendency, Contrast, Correlation, Difference Average, Difference Entropy, Difference Variance, Id, ldm, Idmn, Idn, Imc1, Imc2, Inverse Variance, Joint Average, Joint Energy, Joint Entropy, MCC, Maximum Probability, Sum Average, Sum Entropy, Sum Squares<br>**GLDM:** Dependence Entropy, Dependence Non Uniformity, Dependence Non Uniformity Normalized, Dependence Variance, Gray Level Non Uniformity, Gray Level Variance, High Gray Level Run Emphasis, Large Dependence Emphasis, Large Dependence High Gray Level Emphasis, Large Dependence Low Gray Level Emphasis, Low Gray Level Emphasis, Small Dependence Emphasis, Small Dependence High Gray Level Emphasis, Small Dependence Low Gray Level Emphasis<br>**GLRLM:** Gray Level Non Uniformity, Gray Level Non Uniformity Normalized, Gray Level Variance, High Gray Level Run Emphasis, Long Run Emphasis, Long Run High Gray Level Emphasis, Long Run Low Gray Level Emphasis, Low Gray Level Run Emphasis, Run Entropy, Run Length Non Uniformity, Run Length Non Uniformity Normalized, Run Percentage, Run Variance, Short Run Emphasis, Short Run High Gray Level Emphasis, Short Run Low Gray Level Emphasis<br>**GLSZM:** Gray Level Non Uniformity, Gray Level Non Uniformity Normalized, Gray Level Variance, High Gray Level Zone Emphasis, Large Area Emphasis, Large Area High Gray Level Emphasis, Large Area Low Gray Level Emphasis, Low Gray Level Zone Emphasis, Size Zone Non Uniformity, Size Zone Non Uniformity Normalized, Small Area Emphasis, Small Area High Gray Level Emphasis, Small Area Low Gray Level Emphasis, Zone Entropy, Zone Percentage, Zone Variance<br>**NGTDM:** Busyness, Coarseness, Complexity, Contrast, Strength

GLCM gray level co-occurrence matrix, GLDM = Gray Level Dependence Matrix, GLRLM = Gray Level Run Length Matrix, GLSZM = Gray Level Size Zone Matrix, NGTDM = Neighboring Gray Tone Difference Matrix.

4. Description of the traditional machine learning algorithms

Multilayer perceptron (MLP). The architecture of the MLP is completely defined by an input layer, one or more hidden layers, and an output layer. The input data is processed by the MLP in a forward direction, passing through each single layer. The training of the network is accomplished based on a supervised learning technique (backpropagation) that requires given input-output data pairs.

Adaptive boosting (AdaBoost). AdaBoost is an ensemble learning method and can be used in conjunction with many other types of learning algorithms to improve performance. The output of the other learning algorithms ('weak learners') is combined into a weighted sum that represents the final output of the boosted classifier. AdaBoost is adaptive in the sense that subsequent weak learners are tweaked in favor of those instances misclassified by previous classifiers. AdaBoost typically combines weak learners (such as decision stumps) but can also effectively combine strong learners (such as deep decision trees).

Random forest. The random forest is an ensemble method composed of many smaller models. The classification and prediction is achieved by combining the outputs of these smaller models which are usually classification and regression trees (CART). CART operates based on a repeated partitioning of the input data in order to estimate the conditional distribution of a response (output class) for a given set of feature variables. The algorithm implements a binary decision tree where every single feature of the input is considered as a candidate for the split. Binary decision trees are nonlinear multistage classifiers. This classification system operates by searching a tree-based decision system. The trees are combined to a forest based on bagging. To avoid overfitting, each model is fitted only to a sample of the same size as the original input data but selected with replacement. This sample technique is known as the bootstrap sample.

5. Model optimization

Supplementary Table S5. List of optimized hyperparameters.

Model	Optimized Hyperparameters
Multilayer perceptron	Number of hidden layers, number of neurons, learning rate (selected 0.5; optimization range: 0.1 – 0.3), momentum (selected 0.2; optimization range: 0.1 – 0.3)
Adaptive boosting	Maximum depth of the tree (unlimited), numer of trees in the random forest (selected 100, optimization range: 10 – 1000)
Random forest	Classifier (selected J48, optimization range: all available classifiers)
1D-CNN	Number of hidden layers, number of filters, kernel size, stride, activation functions, dropout rates, loss functions, learning rate, optimizer
LSTM	Number of layers and units, activation functions, dropout rates, learning rate, optimizer

CNN = Convolutional Neural Networks; LSTM = Long short-term memory

6. Description of the 1-dimensional Convolutional Neural Network (1D-CNN)

Supplementary Table S6. Parameters of one-dimensional convolutional neural network (1D-CNN).

Type	Input size	No. of Filters	Kernel size	Stride	Padding	Activation
Input	74, 1					
1D Convolution Layer	74, 1	8	2	1	same	ReLU
Max Pooling Layer	74, 8		2	1	same	
1D Convolution Layer	74, 8	8	3	1	same	ReLU
Drop out (rate 0.2)	74, 8					
Max Pooling Layer	74, 8		2	1	same	
1D Convolution Layer	74, 8	8	3	1	same	ReLU
Drop out (rate 0.2)	74, 8					
Max Pooling Layer	74, 8		2	2	same	
Flatten Layer	37, 8					
Dense layer	296					ReLU
Drop out (rate 0.2)	296					
Dense layer	296					Sigmoid

ReLU = rectified linear unit.

7. Model Performance Testing

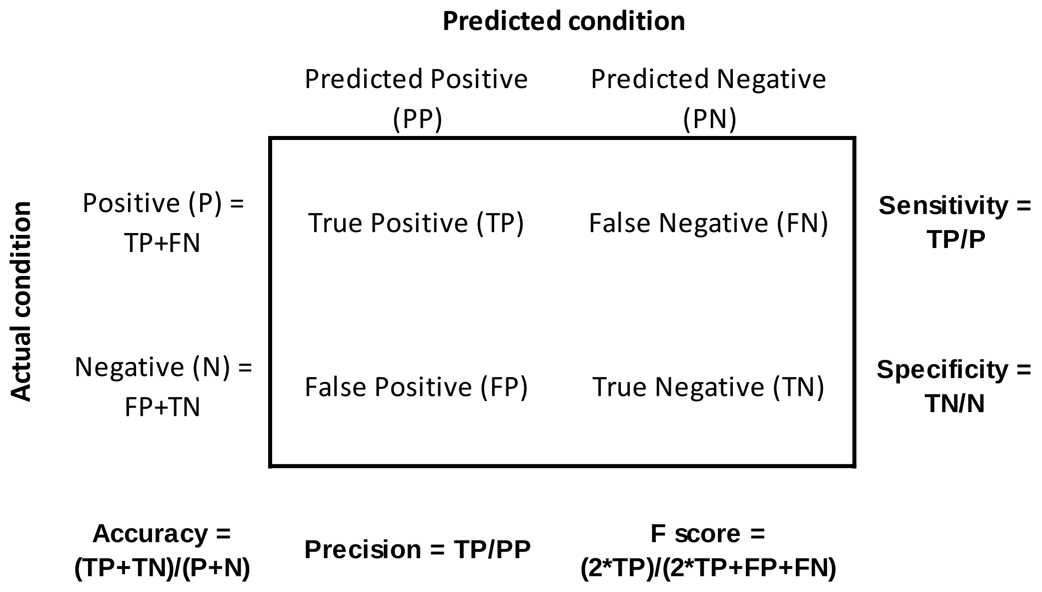

Supplementary Figure S2: Calculation of the confusion matrix-derived metrics.

Supplementary Results

Independent Internal Testing

	Actual	ABoost wt	ABoost mut	MLP wt	MLP mut	RF wt	RF mut	CNN wt	CNN mut	LSTM wt	LSTM mut
**Clinical MRI**	**wt**	8	4	9	3	9	3	9	3	10	2
	**mut**	1	3	1	3	1	3	1	3	1	3
**Oxygen Metabolism**	**wt**	10	2	10	2	9	3	9	3	10	2
	**mut**	1	3	1	3	1	3	2	2	0	4
**Vascular Architecture Mapping**	**wt**	8	4	10	2	10	2	11	1	10	2
	**mut**	1	3	1	3	1	3	1	3	1	3
**Oxygen Metabolism & VAM**	**wt**	9	3	10	2	11	1	9	3	9	3
	**mut**	1	3	0	4	1	3	2	2	1	3

Independent External Testing

	Actual	ABoost wt	ABoost mut	MLP wt	MLP mut	RF wt	RF mut	CNN wt	CNN mut	LSTM wt	LSTM mut
**Clinical MRI**	**wt**	16	5	18	3	18	3	16	5	17	4
	**mut**	1	11	5	7	4	8	4	8	4	8
**Oxygen Metabolism**	**wt**	17	4	15	6	17	4	15	6	18	3
	**mut**	5	7	7	5	7	5	7	5	7	5
**Vascular Architecture Mapping**	**wt**	17	4	18	3	18	3	14	7	17	4
	**mut**	8	4	6	6	6	6	5	7	6	6
**Oxygen Metabolism & VAM**	**wt**	16	5	19	2	21	0	14	7	19	2
	**mut**	8	4	5	7	9	3	6	6	7	5

← Predicted

↑ Actual

Supplementary Figure S3: Confusion matrices for the independent internal and the independent external testing. ABoost = adaptive boosting; MLP = multilayer perceptron; RF = random forest; CNN = Convolutional Neural Networks; LSTM = Long short-term memory; wt = IDH wild-type glioma, mut = IDH mutated glioma.

References

1. Carré, A.; Klausner, G.; Edjlali, M.; Lerousseau, M.; Briend-Diop, J.; Sun, R.; Ammari, S.; Reuzé, S.; Alvarez Andres, E.; Estienne, T.; et al. Standardization of brain MR images across machines and protocols: bridging the gap for MRI-based radiomics. *Sci. Rep.* **2020**, *10*, 12340, doi:10.1038/s41598-020-69298-z.

2. Collewet, G.; Strzelecki, M.; Mariette, F. Influence of MRI acquisition protocols and image intensity normalization methods on texture classification. *Magn. Reson. Imaging* **2004**, *22*, 81–91, doi:10.1016/j.mri.2003.09.001.

3. Shafiq-Ul-Hassan, M.; Zhang, G.G.; Latifi, K.; Ullah, G.; Hunt, D.C.; Balagurunathan, Y.; Abdalah, M.A.; Schabath, M.B.; Goldgof, D.G.; Mackin, D.; et al. Intrinsic dependencies of CT radiomic features on voxel size and number of gray levels. *Med. Phys.* **2017**, *44*, 1050–1062, doi:10.1002/mp.12123.

4. van Griethuysen, J.J.M.; Fedorov, A.; Parmar, C.; Hosny, A.; Aucoin, N.; Narayan, V.; Beets-Tan, R.G.H.; Fillion-Robin, J.-C.; Pieper, S.; Aerts, H.J.W.L. Computational Radiomics System to Decode the Radiographic Phenotype. *Cancer Res.* **2017**, *77*, e104–e107, doi:10.1158/0008-5472.CAN-17-0339.

5. Zwanenburg, A.; Vallières, M.; Abdalah, M.A.; Aerts, H.J.W.L.; Andrearczyk, V.; Apte, A.; Ashrafinia, S.; Bakas, S.; Beukinga, R.J.; Boellaard, R.; et al. The Image Biomarker Standardization Initiative: Standardized Quantitative Radiomics for High-Throughput Image-based Phenotyping. *Radiology* **2020**, *295*, 328–338, doi:10.1148/radiol.2020191145.

6. Haralick, R.M.; Shanmugam, K.; Dinstein, I. Textural Features for Image Classification. *IEEE Trans. Syst. Man. Cybern.* **1973**, *SMC-3*, 610–621, doi:10.1109/TSMC.1973.4309314.

7. Sun, C.; Wee, W.G. Neighboring gray level dependence matrix for texture classification. *Comput. Vision, Graph. Image Process.* **1983**, *23*, 341–352, doi:10.1016/0734-189X(83)90032-4.

8. Galloway, M.M. Texture analysis using gray level run lengths. *Comput. Graph. Image Process.* **1975**, *4*, 172–179, doi:10.1016/S0146-664X(75)80008-6.

9. THIBAULT, G.; FERTIL, B.; NAVARRO, C.; PEREIRA, S.; CAU, P.; LEVY, N.; SEQUEIRA, J.; MARI, J.-L. SHAPE AND TEXTURE INDEXES APPLICATION TO CELL NUCLEI CLASSIFICATION. *Int. J. Pattern Recognit. Artif. Intell.* **2013**, *27*, 1357002, doi:10.1142/S0218001413570024.

10. Amadasun, M.; King, R. Textural features corresponding to textural properties. *IEEE Trans. Syst. Man. Cybern.* **1989**, *19*, 1264–1274, doi:10.1109/21.44046.
